# Event Characteristics and Team Adaptation in Extreme Contexts: Evidence from an Antarctic Summer Campaign

**DOI:** 10.1177/10596011241287945

**Published:** 2024-10-03

**Authors:** Pedro Marques-Quinteiro, Jan B. Schmutz, Mirko Antino, Walter J. Eppich, M. Travis Maynard

**Affiliations:** 1 Lusofona University, Intrepid Lab, Lisbon, Portugal; 2 CETRAD, Oporto, Portugal; 3 Department of Psychology, University of Zürich, Zürich, Switzerland; 4 Departamento de Psicobiología y Metodología en Ciencias del Comportamiento, Universidad Complutense de Madrid, Madrid, España; 5 Faculty of Medicine, Dentistry and Health Sciences, The University of Melbourne, VIC, Australia; 6 3447 Colorado State University, Fort Collins, CO, USA

**Keywords:** team adaptation, team processes, team performance, extreme environments

## Abstract

Team adaptation is particularly impactful within extreme and isolated environments, where sudden and abrupt events drastically challenge effective teamwork. To advance the team adaptation literature, we examined how event characteristics influence the relationship between team adaptation processes and team adaptive performance. To do so, we conducted an on-site, multi-study research using sequential explanatory mixed methods and a retrospective event history approach. The first study (based on a quantitative multilevel methodology) was designed to understand how the characteristics of the events influenced team adaptation processes and team adaptive performance (we collected data of 86 events described by 56 informants nested within 21 teams) during one Antarctic Summer Campaign at the South Shetland Islands Archipelago, Antarctica. The second study, based on qualitative methodology focused on thematic analysis, was designed to obtain a detailed description of the relationship between adaptation triggers and team adaptation (we collected data from 20 semi-structured interviews). Overall, our findings highlight that different team processes are significant in shaping perceptions of team adaptive performance, making the modification of transition and interpersonal processes the most critical. We additionally show how these relationships are moderated by the characteristics of adaptation triggers. We discuss the implications of these findings for teams within extreme environments and beyond.

## Introduction

A cascade of system malfunctions on the way to the moon, shifting wind directions requiring revised fire suppression efforts, and conflicts with teammates during an around-the-world sailing expedition: these examples illustrate the diverse challenges teams face in extreme environments. Such environments are characterized by isolation from the outside world, confinement to restricted living spaces, and exposure to extreme conditions outside the control of individual team members or the organization (Hannah et al., 2009). Additionally, these inherent challenges amplify the frequency and impact of events that require significant team adaptation, defined as how teams modify team processes (i.e., action, transition, and interpersonal) in response to adaptation triggers that emerge during missions and necessitate adaptation (Maynard et al., 2015; Schmutz et al., 2023).

**Figure 1.**
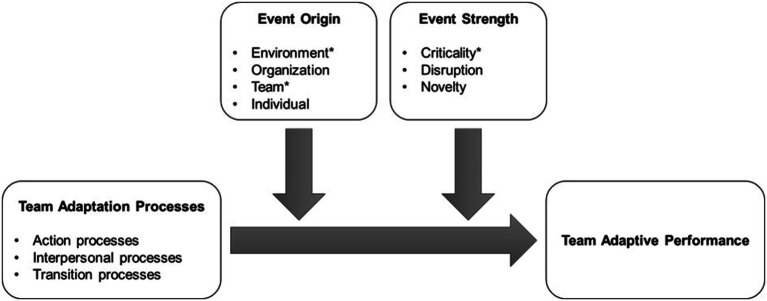
The general research model for Study 1 and Study 2. The * highlights the event characteristics that were considered in Study 1.

These adaptation triggers, like those examples mentioned above, can also be viewed as ‘events’, terms which we use interchangeably here. Research on team adaptation in response to critical events has consistently shown the importance of input factors such as training and team member characteristics, as well as mediating factors such as team processes to achieve effective teamwork (e.g. Burke et al., 2006; Li et al., 2023; Rico et al., 2019; Uitdewilligen et al., 2023). However, while team processes have been examined within the team adaptation literature, we lack robust consideration of the different types of team processes detailed within the team effectiveness literature, that is, “team members’ interdependent acts that convert inputs to outcomes through cognitive, verbal, and behavioral activities directed toward organizing taskwork to achieve collective goals” (Marks et al., 2001, p. 357). In response, this paper considers team processes within the team adaptation nomological network put forth by Maynard et al. (2015). We explore various types of processes within a single study (action, transition, and interpersonal), and examine whether different processes play different roles in shaping team adaptive performance in response to key events. For this study, we define event characteristics as those bound in space and time that break people out of routines and challenge performance (Morgeson & DeRue, 2006). Thus, this study examines if event characteristics shape the extent to which certain team processes are more or less critical to teams’ adaptive performance (e.g. Maynard et al., 2015). By treating events and adaptation triggers interchangeably, we expand the available theoretical lenses to inform this work. By investigating the relationship between these event characteristics and team’s adaptive performance, illustrated by Figure 1, we integrate Event Systems Theory (Morgeson et al., 2015) and the team adaptation nomological network (Maynard et al., 2015) and use an event-level analysis approach.

We see four main contributions of our work. First, we address a gap related to the insufficient examination of the underlying characteristics of context-specific events and their significance to teamwork effectiveness overall (e.g., Christian et al., 2017). Specifically, we address the lack of field studies that explicitly focus on team adaptation in response to critical events in the wild, the consideration of how specific elements of the teams’ work context relate to team adaptation (i.e., triggering events), and deeper reflections of how all types of team processes (including interpersonal processes which have not been extensively examined within the team adaptation literature) shape perceptions of team adaptative performance in real teams in the field (Burke et al., 2006; Christian et al., 2017; Maynard et al., 2015, 2021). Second, we integrate Event Systems Theory (Morgeson et al., 2015) within the team adaptation literature, which provides a new theoretical lens to test and expand Maynard et al. (2015) theorizing about how context-specific event characteristics are boundary conditions to the adaptation of team processes leading to team adaptive performance. Third, our work contributes to recent initiatives from space agencies such as the National Aeronautics and Space Administration (NASA) and the European Space Agency (ESA), which have advocated for the use of space analog environments such as Antarctica to study the factors that enable team adaptation and mitigate mission dangers during short and long duration space missions (Landon et al., 2018; Palinkas & Suedfeld, 2021). Fourth, our research also provides generalizable insights to help more traditional teams because most teams across sectors at some point will face extreme events that demand team adaptation (e.g., the 2007 financial crisis; the COVID-19 crisis).

We conducted an on-site, multi-study research project using sequential explanatory mixed methods (Creswell, 2018) and a retrospective event history approach (Glick et al., 1990). We utilize these approaches because research examining teamwork in extreme environments is best pursued in real settings (Bell et al., 2018; Kennedy et al., 2023). Additionally, on-site data collection reduces reconstruction bias, which becomes more pronounced over time as participants recall the details of stressful situations. This aspect is a key issue in our theoretical approach since events are our primary focus and our unit of analysis (Ohly et al., 2010). Hence, we collected and analyzed quantitative and qualitative data from individuals and teams working during the 2020 summer season at the South Shetland Islands Archipelago, Antarctica. Our sequential explanatory strategy initially emphasizes quantitative data analysis to provide a general understanding of the research problem, followed by qualitative data analysis to refine and explain statistical results by exploring participants’ views in more depth (Creswell, 2018). This approach is particularly useful when researchers first seek to test specific hypotheses and then gather richer information that provides context and clarifies nuances relevant to the unique context. Especially in extreme environments, studies designed to describe and understand behavior within these contexts have often been neglected in team research (Bell et al., 2018).

## Theoretical Background

Working in extreme environments can result in overwhelming physical, psychological, or material consequences to team and organization members, beyond what we observe in business and education (Hannah et al., 2009; Schmutz et al., 2023). Teams in extreme environments face stressors that traditional teams rarely face, including isolation from the outside world with limited communication and resources, and people must manage with available supplies. Teams face confinement, limited privacy, and blurred lines between work and leisure. The hostile environment itself represents a significant stressor, and as a result, successful performance often requires significant team adaptation to respond to the events that happen during the missions (e.g., Landon et al., 2018; Mesmer-Magnus et al., 2016; Salas et al., 2015).

Surprisingly, our understanding of team adaptation in extreme environments remains limited; we lack clarity about how team adaptation happens beyond lab and traditional organizational environments (Maynard et al., 2021; Mesmer-Magnus et al., 2016; Salas et al., 2015). This is, in part, due to the inherently small sample sizes in these contexts, generalizability concerns, as well as difficult access to research sites and populations (Bell et al., 2018). While some have studied team adaptation in controlled settings (e.g., Parker et al., 2018; Santos et al., 2021), others argue that we must study teams in extreme environments to gain deeper understanding of what team adaptation is and how it happens in the wild (Maynard et al., 2021; Schmutz et al., 2023). Teams in extreme environments constantly adapt to mitigate risks and overcome challenges, making such settings ideal for studying effective team dynamics. What is more, examining interactions in extreme environments provides insights into how teams adapt to emergent events and under prolonged strain, which is more challenging to replicate within laboratory settings (Hällgren et al., 2018; Maynard et al., 2021).

While larger sample size studies are methodologically sound, our literature often neglects numerous teams and potential team phenomena by predominantly favoring such studies (Kennedy et al., 2023). However, small sample team research holds considerable value. Gersick’s (1991) seminal work on punctuated equilibrium, Cherry-Garrard’s narration of Scott’s disastrous expedition to the South Pole, and Tempest et al.’s case study of the mount Everest disaster exemplify the profound insights gleaned from single and small team research (Tempest et al., 2007). Moreover, teams in extreme environments qualify as rare sample populations (Christman, 2009) – namely, those in which the size of the population (*N*) is very small, especially in comparison to the broader population (i.e., Antarctica population vs. World’s population). When researching rare populations, small sample sizes are often inevitable. Nonetheless, such samples can still faithfully represent the broader population (Christman, 2009) and provide invaluable insights into complex team dynamics, including phenomena such as a decrease in the number of interaction processes during the third quarter of Antarctica winter-overs, salutogenesis, and ingroup-outgroup dynamics. These insights may outweigh the constraints posed by smaller sample sizes. Furthermore, the inherent limitations of small samples necessitate a methodological shift towards mixed methods approaches. By integrating both quantitative and qualitative methodologies (as we do here), researchers can achieve a more comprehensive analysis, allowing for a deeper exploration of intricate dynamics and theories, such as Event Systems Theory (Kennedy et al., 2023; Morgeson et al., 2015). This blend of methods may not only compensate for the small sample size but also enrich the research findings by providing a multifaceted view of the subject matter.

In the current study, we sought to investigate teamwork in extreme environments and leverage the value of small sample team research within the context of Antarctica, which is the windiest, coldest, and driest continent on Earth. Likewise, Antarctica is dynamic and remote, and life is characterized by isolation, confinement, and extremeness (ICE). These same features have made Antarctica one of the best natural settings to study human behavior in extreme environments because unexpected events are plentiful, offering great opportunities for team adaptation research (Golden et al., 2018; Schmutz et al., 2022). However, beyond consideration of the novelty of the context in which we conduct our research, we also expand upon the team adaptation literature (Maynard et al., 2015) and integrate the Event Systems Theory (EST) (Morgeson et al., 2015) to hypothesize how the nature of adaptation triggers (i.e., the disruptiveness and origin of events in our case, following Morgeson et al., 2015) shapes the relationship between team adaptation processes and perceptions of team adaptive performance in extreme environments.

### Small Groups in Extreme Contexts

Scholarly interest in the study of human collaboration in extreme environments emerged around 75 years ago, following the end of the Second World War (Bell et al., 2018; Palinkas & Suedfeld, 2021). Considerations of extreme environments have increased following the advent of the space era (Burke et al., 2018; Golden et al., 2018). Over this time, researchers and practitioners alike have focused on delineating the factors that characterize an extreme environment, identifying the challenges inherent to such contexts, and unpacking the psychosocial dynamics of those who live and perform in extreme environments such as the polar regions (e.g., Kanki & Gregorich, 1992; Norris et al., 2010). For instance, Hannah et al., (2009), introduced a typology of extreme contexts and suggested that certain dimensions of extreme environments exacerbate the level of extremity to which team leaders must adapt during missions and expeditions. These dimensions include the form of threat (e.g., snow-covered crevices) and the magnitude and probability of the consequences arising from those threats (e.g., severe injuries). The negative impact of these dimensions is amplified under increased threat duration and task complexity, and attenuated if psychological, social, and organizational resources are available (e.g., Schmidt et al., 2004; Wagstaff & Weston, 2014).

Others have built upon these initial considerations of extreme context characteristics, with Orasanu and Lieberman (2011) proposing a categorization of extreme environments that includes normal environments – extreme behavior (e.g., rock climbing; car racing), extreme social environments (e.g., military operations; hostage negotiation), extreme environments – extreme behaviors (e.g., Coast guard rescue; Ocean racing), and isolated, confined, and extreme (ICE) environments (e.g., space; polar expeditions). This work was recently expanded by two other contributions, including an event-based taxonomy of extreme contexts (Hällgren et al., 2018), and the introduction of team extremeness as a continuum construct that includes consideration of both environmental and task factors that contribute to enhanced levels of team extremeness (Schmutz et al., 2023). These contributions allow us to regard Antarctica as an ICE environment (Orasanu & Lieberman, 2011) that is also a risky context of imminent danger (Hällgren et al., 2018), and which has a high extremeness level (Schmutz et al., 2023).

Similarly, researchers have examined factors that appear to hamper team effectiveness within such extreme settings and provided evidence that cultural differences, the formation of subgroups, and the lack of privacy often hinder teamwork effectiveness (Leon et al., 2011; Palinkas & Suedfeld, 2021). Leadership has also been pinpointed as another critical element of effective teamwork in extreme environments because it sustains the development, conservation, and restoration of team cohesion and team trust during missions and can result in individual team members feeling supported (Burke et al., 2018; Wood et al., 2005). Research about teamwork in extreme environments has also highlighted the importance of team composition in mitigating conflict that emerges from such factors as subgroup formation due to high diversity and minimal levels of shared values. Unsurprisingly, empirical studies have found high levels of stress can have negative impacts on communication, decision-making, and performance, which can be mitigated through coping strategies such as social support, and problem-focused coping (Leon & Sandal, 2003; Sandal et al., 1999).

### Team Adaptation Processes

Over the last two decades, research has systematically demonstrated many positive impacts of team processes on teamwork outcomes while also highlighting that team process categories have distinct and unique effects on adaptive performance and team adaptation outcomes (Christian et al., 2017; Mathieu et al., 2019). For example, Entin and Serfaty (1999) showed that teams who learned when to shift between explicit and implicit coordination strategies outperformed those who did not learn how to do it when facing sudden increases in task complexity. Additionally, LePine (2005) tested how command and control teams adapted to incremental disruptions in the team communication network. More recently, Burke et al. (2018) found that team leaders enable role structure adaptations by changing who oversees the coordination of specific activities so that teams may adapt their processes to respond to predicted and unpredicted mission changes in Antarctica. Further, Jonassen and Hollnagel (2019) found that maintaining an open and flexible work structure encourages the involvement of individual team members in problem-solving processes and enables adaptation within offshore oil platforms. Overall, these studies show that the ability to change team processes in response to events that require adaptation is positively related with team adaptation outcomes such as performance and learning under extreme circumstances.

In part, some of these more recent empirical examinations of team adaptation may be attributable to conceptual arguments made in the literature emphasizing the need for a greater consideration of team processes within the team adaptation nomological network. Maynard and colleagues (2015) introduced their perspective which argued that team process adaptation is a key mediating mechanism that should be examined within deeper examinations of the relationships between team adaptation triggers and team adaptive performance. In fact, Maynard and colleagues argued that team adaptation occurs through the successful modification of team processes. As a point of emphasis here, these authors did not suggest that team processes are what should be examined, instead they argued that the emphasis should be on how team processes are adapted and altered. This differs from prior work which has either only examined processes in general and not whether such processes were adapted when responding to a challenge. Likewise, while prior work has considered certain types of processes, Maynard and colleagues (2015) suggested that researchers need to more formally examine all types of team processes to better understand whether certain processes are more or less salient in response (or in anticipation) to adaptation triggers. To accomplish this goal, Maynard and colleagues (2015) suggest that research needs to more fully examine the characteristics of the trigger(s) being faced by the team to gain a deeper understanding of which team processes need to be adapted to enhance team adaptive outcomes.

Team processes are a central part of almost every team effectiveness framework (Marks et al., 2001; Mathieu et al., 2019) and have been studied extensively within the team effectiveness literature. Given the attention given to team processes, it is not surprising that numerous works have tried to establish a framework or typology of team processes (e.g., Mathieu et al., 2020; Maynard et al., 2015). That said, the work of Marks and colleagues (2001) has gained substantial traction over the past decades, and Maynard and colleagues (2015) used this typology of team processes in their conceptual work. Thus, we also use this typology in the current study. According to Marks and colleagues (2001) there are three overarching types of team processes: action, transition, and interpersonal. As suggested by Marks and colleagues, *action processes* included members focusing on task accomplishment, monitoring progress/systems, coordination, and monitoring and backing up teammates. In contrast, *transition processes* include members engaging in activities such as mission analysis, planning goal specification, and formulating strategies.

To date, when processes have been considered within the team adaptation literature, they have typically been either action or transition processes (e.g. LePine, 2005). However, Marks and colleagues (2001) also highlighted *interpersonal processes* which include conflict management, motivation, and confidence building, and affect management and to date, the team adaptation literature has not fully examined this category of team processes extensively.

Again, in contrast to only looking at these types of team processes within studies of team adaptation, Maynard and colleagues (2015) advocated that researchers need to consider whether teams need to adapt these processes. For instance, do teams need to change how they communicate when faced with team adaptation triggers? Likewise, do the changes in communication processes differ depending on the type of trigger and the triggers’ inherent characteristics? Maynard and colleagues theorize answers to these questions in their conceptual work; we seek to examine them here. To date, an examination of the relationships suggested by Maynard and colleagues (2015) has not been conducted and therefore, there is limited empirical evidence regarding the relationships between the adaptation of team processes with team adaptive performance. That said, evidence suggests that each process category is helpful for team adaptive performance (Mathieu et al., 2020). Therefore, here we propose formal hypotheses suggesting that adapting each type of team process should positively impact perceptions of team adaptive performance. We hypothesize that:

Hypothesis 1
The modification of action (*Hypothesis 1a*), transition (*Hypothesis 1b*), and interpersonal (*Hypothesis 1c*) processes is positively related with team members’ perceptions of team adaptive performance.

### Event trigger Characteristics

Some factors likely moderate the relationship between the adaptation of team processes and team adaptive performance, namely adaptation triggers and their characteristics (Georganta et al., 2019; Kennedy & Maynard, 2017). The team adaptation literature refers to adaptation triggers as *events* or changes that occur at any organizational level and that, once recognized by team members, prompt teams to modify their processes to complete their tasks successfully (Burke et al., 2006). Furthermore, adaptation triggers’ characteristics should define what relevant team processes ought to change, and the extent to which those changes lead to positive team adaptive outcomes (Kennedy & Maynard, 2017). For example, Christian et al. (2017) examined the interplay between team cognitions, adaptation triggers, and specific team processes such as communication. Yet, the literature has not yet investigated differential effects of various triggers on the adaptation of different team processes. This nuance is important to the team adaptation literature because we need to better understand how team adaptive performance relies on changing these different types of team processes to respond to specific events and their characteristics in the first place (Kennedy & Maynard, 2017).

Preliminary research has suggested that the extent to which teams adapt team processes in response to unexpected events that threaten performance is moderated by event trigger characteristics such as the stressfulness of the event (Entin & Serfaty, 1999), and goal difficulty (LePine, 2005). However, even with this preliminary examination of triggers, many authors have recommended more work, specifically calling for research to examine the impact of trigger characteristics on team adaptation, and specifically how such trigger characteristics serve as moderators of the process-performance relationship (e.g., Christian et al., 2017; Kennedy & Maynard, 2017). In this study, we expect event trigger characteristics to play a major role in moderating how adapting the different types of team processes will result in positive or negative perceptions of team adaptive performance (Maynard et al., 2015).

While there have been various attempts to categorize events and pinpoint the features of an event that alter behavior, Morgeson et al. (2015) offer the most prominent with their EST in which they highlight three event components: (1) *event strength* (including novelty, disruption, and criticality dimensions); (2) *event space* (including spatial direction, origin, spatial proximity, and spatial dispersion), and (3) *event time* (including when an event occurs, the duration of its impact, and the evolution of event strength). Although it is difficult to align one’s measurement completely with the theorized dimensions in the field, in the current study, we focus on the criticality and the origin of adaptation triggers because: (a) scholars generally agree on the importance and stability of these two trigger characteristics across contexts (e.g., Christian et al., 2017; Maynard et al., 2015; Morgeson & DeRue, 2006); (b) empirical examinations of how they influence team adaptation have been limited, and (c) it was not possible to implement the longitudinal research design that would be ideal to consider event time characteristics (Morgeson et al., 2015). Hence, in the following sections, we elaborate on how event trigger criticality moderates the relationship between team adaptation processes and perceptions of team adaptive performance, and how event trigger origin adds to that relationship.

### Event Trigger Criticality

As defined by Morgeson and DeRue (2006), the criticality dimension of an event strength is defined as “the degree to which an event is important, essential, or a priority” (p. 273). The event strength dimension recognizes that not all events are created equal, therefore not all events have the same effect on the team and require the same amount of attention by team members and leaders. In fact, more critical events require more attention and resource allocation than less critical events (e.g., Gersick & Hackman, 1990). Likewise, as Morgeson and DeRue (2006) articulated, more critical events become a greater part of the team’s focus until event resolution.

We contend that with more critical events, teams must change their processes to enable adaptive performance. This is partly because more critical events require prioritizing a team’s response to an event (Gersick & Hackman, 1990). Therefore, we can envision that as teams face more critical events, it becomes more important that they align and adapt their action, transition, and interpersonal processes to the changing situation to achieve necessary levels of team adaptive performance. This is like the relationship examined by Chen et al. (2022), who found support for the event criticality of COVID-19 positively moderating the relationship between a firm’s IT-business alignment and firm decision speed and quality. As events become more critical, the relationship between the adaptation of various team processes and adaptive performances should be magnified.

Hypothesis 2
Event trigger criticality moderates the relationship between team adaptation processes and perceptions of team adaptive performance, such that event triggers with higher criticality will enhance the relationship between action* (Hypothesis 2a), *transition* (Hypothesis 2b), *and interpersonal processes (Hypothesis 2c) and perceptions of team adaptive performance, to a greater extent than events with lower criticality.

### Event Trigger Origin

Within the team adaptation literature, a growing consensus highlights that the origin of adaptation triggers moderates the relationship between changes in team adaptation processes and team adaptive performance differently, and that this relationship also has implications for other adaptation trigger characteristics (Gersick & Hackman, 1990; Kennedy & Maynard, 2017). As an example, Maynard et al. (2015) theorized that the specific type(s) of team processes that should be adapted to enable performance may differ depending on whether the event trigger is taskwork or teamwork oriented, and the extent of perceived severity. Along the same lines, Christian et al. (2017) performed a meta-analysis in which event triggers were classified as *internal* (defined as any changes in roles, membership, rewards, or structural form of the team), and *external* (defined as any changes in the collective task environment, including changes in situational contingencies and the occurrence of non-routine events). Christian et al. (2017) found that adaptive stimulus origin moderated the nuanced relationship between team processes and team adaptive performance overall. The relationship between team processes and team adaptive performance was stronger for external triggers when processes included action processes like coordination and stimulus specific actions. Although the meta-analysis did not reveal a significant moderation effect of trigger origin on the relationship between plan formulation (as a specific transition process) and adaptive performance, the mean corrected correlation between plan formulation and adaptive performance was stronger for internal triggers, rather than external. Additionally, no empirical results were reported on interpersonal processes, thus pointing to a gap that the team adaptation literature has yet to examine. Overall, these results empirically support the idea that the event trigger origin has different implications in terms of which team processes need to change in the face of such a trigger, and how that change relates to team adaptive performance.

Unfortunately, while Maynard et al. (2015) were among the first to specifically argue that different processes need to be adapted in the face of different types of triggers, relatively little empirical attention has delineated these relationships. In fact, the meta-analysis conducted by Christian et al. (2017) is one of the first to investigate such moderation relationships. However, since the meta-analysis largely pulls from studies that have been conducted within more *traditional* team environments, it remains unclear as to whether these relationships remain within extreme contexts, which is another contribution of the current study. Likewise, given that the literature has provided more emphasis on action and to a lesser extent transition processes with almost no attention to interpersonal processes, our understanding of the entirety of the team process adaptation – team adaptive performance relationship is incomplete.

Viewed broadly, EST regards event origin as the “hierarchical level at which an event occurs” (Morgeson et al., 2015, p. 525), which includes environments, organizations, teams, and individuals. Where the event happens within the organizational ecosystem will likely moderate the relationship between other event trigger characteristics, processes, and their outcomes, and as suggested within the EST theory, event origin likely either buffers or amplifies any moderating effects by additional event trigger characteristics. Hence, the moderation of trigger event characteristics on the relationship between team adaptation processes and team adaptive performance should be stronger for events with higher criticality and we suggest that this moderation will be even more pronounced for events that happen higher in the organizational ecosystem (Morgeson et al., 2015). As an example, environment level events which are seen with higher criticality by those directly affected by it (e.g., a snowstorm; a stock-market crash), should have a stronger moderation effect on the relationship between team adaptation processes and team adaptive performance, compared to organization level events (e.g., a temporary power cut), or team (e.g., a piece of equipment breaks) levels and with lower perceived criticality by those directly affected by it. These nuanced relationships are apt to happen as hypothesized because of the nested structure of organizational systems. The higher the level at which an event happens, the more implications it will have for the dynamics unfolding at lower levels, whereas the opposite is less likely to happen (Morgeson et al., 2015). Hence, we anticipate that team adaptation processes enable team adaptive performance, and that event trigger criticality and event trigger origin will moderate this relationship (Christian et al., 2017; Maynard et al., 2015). Team adaptation processes will be the most important when teams face highly critical events that happen higher in the organization. We hypothesize that:

Hypothesis 3
Event trigger criticality and event trigger origin moderate the relationship between team adaptation processes and perceptions of team adaptive performance, such that event triggers with higher criticality and which originate at higher levels will more strongly moderate the relationship between action* (Hypothesis 3a), *transition* (Hypothesis 3b), *and interpersonal processes (Hypothesis 3c) and perceptions of team adaptive performance, than events with lower criticality and originating at lower levels.

## Methods - Quantitative Study 1

This research was approved by the National Authority for Environmental Protection and the first author’s former University Ethical Committee (ref. I-028-11–2019).

### Research Context and Participants

The Antarctic environment is highly dynamic, characterized by tremendous ambiguity and complexity that pose extraordinary challenges for humans living and working there. Therefore, this setting is ideal for examining relevant group phenomena such as team adaptation in extreme environments (Schmutz et al., 2023). King George Island, where our data was collected, is the largest island in the South Shetland Islands archipelago. The average temperature during summer ranges between −1–4 degrees Celsius (30–39 degrees Fahrenheit) and the South Peninsula, where most of the research stations are, is ice free. The weather conditions at King George Island are extremely volatile, which often makes outdoor work impossible.

Our study participants included scientists, civilian and military staff, managers, and builders who were working in the South Shetland Islands archipelago during February 2020. In the summer season, science teams of 2–5 people visit the stations to conduct a research project, lasting from two weeks to three months. Most research projects involve field work, and cover domains from the physical, natural, and social sciences. Staff include people who typically remain on the island between 1-3 months. Typically, staff teams consist of 6–14 people who run research stations and provide logistic support to scientists. Typical job descriptions for staff include boat driver, diver, pilot, alpine guide, cook, electrician, carpenter, or mechanic. Finally, the management team at each station bears responsibility for each resident of the station. Management teams consist of 2–4 people. As such, managers make decisions about safety issues (e.g., deciding whether researchers may leave the station) and allocate station resources across the various science teams. Station management ensures safety of the teams, provides information about when to go where, and coordinates the available experienced divers, boat drivers or glaciologists who know the environment and can accompany teams on field trips. Finally, builders are construction personnel involved in the construction and repair of station infrastructure.

### Sampling and Data Collection Approach

Antarctica has no permanent human settlement and its average working population during the summer months (December to February) can reach up to 5000 people, of which around 75% are scientists and the remaining 25% are staff, managers, and builders (Scientific Committee on Antarctic Research SCAR, 2024). In a world of 7.8 billion human beings, that is 0.00,006% of the world’s population. Hence, besides the uniqueness of the Antarctic context and its importance to extend our understanding of team adaptation in extreme environments, those people working in Antarctica can be regarded as a rare population (Christman, 2009). Rare populations are those in which the size of the population (*N*) is very small, especially in comparison to the broader population (i.e., Antarctica population vs. World’s population). In the specific case of rare populations, although it is to be expected that the sample size of research studies is small, such samples are still representative of the broader population. Following Christman (2009), sampling methods for rare populations where the individuals with the unique feature of interest are easily identified (i.e., working in Antarctica, in our case) can be performed free of selection risks. This means that any individual from the target population can be selected to take the survey, without risk of selecting individuals that are not part of the target population (e.g., inviting a tourist to take on the survey).

For the surveys, we applied a retrospective event history approach and invited participants to recall events that happened within one week prior to survey completion (Glick et al., 1990; Spector, 2019). Event-based approaches are best suited for field studies in which events are central to the research objective (Morgeson et al., 2015), and because of their positive impact on reconstruction bias (e.g., Ohly et al., 2010). We did not inquire about general team functioning; instead, we evaluated specific events according to our research goal. We always asked participants to name their teams so we could cluster them in the analysis. The first and second authors interacted with various teams at different stations on the island and in transit to and from Antarctica. They approached potential research participants directly in the field and invited them to complete one survey, either via paper/pencil format or online on an iPad or smartphone in English, Spanish, or Portuguese. Original surveys were in English, and were translated to Spanish, and Portuguese by native speakers. Most invited participants agreed to participate and therefore the acceptance rate was high.

Sixty-four individuals completed the survey, but eight were returned incomplete and were removed from the study prior to data analysis. Hence, our analysis included 56 individuals (*N*_team_ = 21, with team size ranging between 2 and 8 individuals). Mean participant age was 44 years (SD = 13); 71% were men. Seventy-two percent of participants were installed at civilian research stations, 18% in ships, 8% in camps, and 2% in huts. 57% were scientists, 22.4% were staff, 11.1% were management, and 9.4% were builders. Regarding the educational background of research participants, 27.8% of respondents had a PhD, 27.8% had a master’s degree, 19.7% had an undergraduate degree, 9.8% had completed high school, and 11.8% reported “other”. For 20.7% of the research participants, it was their first Antarctic expedition, whereas 22.4% had been in Antarctica before once, and 17.2% had been there twice. The study sample includes participants from 13 nationalities, of which 16% are Portuguese, 15% are Spanish, 15% are Chilean, and 54% come from other nations. All data was collected during visits to different research stations in King George Island (i.e., Great Wall Station; Base Professor Julio Escudero; Artigas Station; and Bellingshausen Station).

Our sample includes science, logistic, and management teams. Science teams conducted research projects that often included some form of field data collection, followed by the storing, processing and eventual analysis of data. Examples of research projects’ fields included biology, glaciology, climatology, sociology, architecture, and engineering. Examples of logistic activities included providing assistance and support to science projects (e.g., helping fix equipment; collecting samples; providing guidance or transport), communications, healthcare, cooking, and maintenance and repairing of infrastructures. Finally, managers were responsible for overseeing all Antarctica operations, managing the distribution and use of resources at research stations, and ensuring law and order.

### Measures

Participants were presented with a list of events that were framed as “challenges that can threaten the success of their campaigns.” These events were derived from both a review of the Antarctic literature (e.g., Palinkas & Suedfeld, 2021) and preliminary interviews of experienced scientists and station managers conducted by our research team (see Table 1). In addition to this list of events, participants had the option of reporting other unlisted events. Since the level of analysis in our study were *events*, and although these happened within teams (hence the adoption of a multilevel analytical approach that reflects the nested nature of our data), there were no specific requisites to ensure that participants from the same team reported the same events.

**Table 1.** Events that Were Adaptation Triggers.

	Frequency
Environment	42 (48.8%)
*Climbing to main study site	3 (3.5%)
Deterioration of living conditions	5 (5.8%)
*Dangerous shipment	1 (1.2%)
Weather change	33 (33.7%)
Team	44 (51.2%)
Conflict with other teams or other people	4 (4.7%)
Conflict within the team	2 (2.3%)
*Work overload	1 (1.2%)
*Forgot equipment	1 (1.2%)
Loss or malfunction of technical equipment	2 (2.3%)
*Managing new groups	1 (1.2)
*Change in working conditions	1 (1.2)
Sample or data loss	9 (10.5%)
*Task overlap	1 (1.2)
Technology failure	22 (25.6%)
**Total**	86 (100%)

*Note.* Adaptation triggers highlighted with an asterisk (*) were reported by individuals, in addition to the list of possible adaptation triggers we provided.

Forty percent of participants reported two events, and 60% of the participants reported one event. As detailed in Table 1, 48.8% of events reported by individuals were environmental triggers with the remaining 51.2% of events being team-based triggers. Finally, for each event, participants were also asked to share their perceptions of event criticality, team adaptation processes, and team adaptive performance while trigger origin was classified based on their descriptions.

#### Event Criticality

Event criticality was measured using four items from Maynard and Hakel (1997), including (a) “the challenge was complex,” (b) “the challenge was mentally demanding,” (c) “the challenge required a lot of thought and problem-solving” and (d) “the challenge was difficult” (Cronbach α = .81). Participants provided their level of agreement on a 5-point Likert type scale ranging from 1 (totally disagree) to 5 (totally agree).

#### Event Origin

We categorized event origins that were the most parsimonious and would fit within EST’s event origin dimensions: environment, organization, team, and individual (Morgeson et al., 2015). Of note, even though some event triggers may have originated from individual or organizational events, we could not determine the root trigger of each event. Therefore, we grouped events reported by participants as events which had their origin from within the team (51.2%) or in the environment (42.8%). Our dataset included 86 events that were our adaptation triggers. The two most frequent team events were technology failures (25.6%) and sample or data loss (10.5%). The two most frequent environmental events were weather changes (38.4%) and terrain roughness (3.5%). Table 1 summarizes all events that participants reported. For our analysis, team events were coded as “1” and environment events were coded as “2”.

#### Team Adaptation Processes

Team adaptation processes were measured using the short version of the team processes inventory (10 items; Mathieu et al., 2020). In line with recommendations by Maynard et al. (2015) on how to study team adaptation processes and their contribution to overall team adaptation, participants were asked about the extent to which their teams had to change the way they engaged in action, transition, and interpersonal processes to respond to each of the events that were reported by team members (Maynard et al., 2015). Four items measured action processes (e.g., “coordinated your activities with one another”; Cronbach alpha = .80); 3 items measured transition processes (e.g., “identified the key challenges that you expect to face”; Cronbach alpha = .74), and 3 items measured interpersonal processes (e.g., “dealt with personal conflicts in fair and equitable ways”; Cronbach alpha = .79). Participants gave their answers on a 5-point Likert scale ranging from 1 (totally disagree) to 5 (totally agree).

#### Perceptions of Team Adaptive Performance

Participants were presented with one item asking: “overall, how effective was your team in adjusting and responding to the challenge?” We adopted a single-item measure to keep participants engaged and minimize their burden in an already taxing environment. Since the item is unambiguous, specifically targets team effectiveness of team adaptation processes, and has face validity, the single-item measure can be regarded as adequate and trustworthy (Matthews et al., 2022). The item was rated on a 5- point Likert scale ranging from 1 (not at all effective) to 5 (highly effective).

### Analytical Approach

Data analysis was performed using a multilevel approach. Data was analyzed at the event level, with events nested in teams. The hypotheses were tested using hierarchical linear modeling (HLM). This analytical strategy allowed us to deal with potential non-independence issues, such as in our case, having data of different events for a specific team. Since the use of HLM has been shown to be robust with a minimum of 20 clusters (20 level 2 sample size; Snijders & Bosker, 2012), we therefore tested multiple models following Aguinis et al. (2013) using the lme4 package for the software (R Core Team, 2014).

### Results

Table 2 shows the means, standard deviations, and correlations for all study variables at the event level (correlations were calculated on the within-team centered variables, to account for the non-independence of measures). The three team adaptation processes were positively correlated between themselves, with the highest correlation occurring between action and interpersonal processes, r = .63, *p* < .01, and the lowest correlation occurring between action and transition processes, r = .36, *p* < .01. Team adaptive performance was positively correlated with all team adaptation processes, with the highest correlation being with transition processes adaptation, r = .71, *p* < .01, and the lowest correlation with action processes adaptation, r = .55, *p* < .05. Also, event criticality was positively correlated with transition and interpersonal processes adaptation, r = .23, *p* < .05, and negatively correlated with event origin, r = −.23, *p* < .05.

**Table 2.** Descriptive Statistics and Correlations.

Variable	*M*	*SD*	1	2	3	4	5
1. Action processes adaptation	3.71	0.97	1				
2. Transition processes adaptation	3.76	0.99	.36**	1			
3. Interpersonal processes adaptation	3.80	1.06	.63**	.47**	1		
4. Event origin	1.49	0.50	−.03	−.07	−.05	1	
5. Event criticality	3.06	1.03	.02	.23*	.23*	−.23*	1
6. Perceptions of team adaptive performance	4.09	0.97	.55**	.58**	.71**	−.03	−.14

*Note. N* = 86 events. *p < .05; **p < .01, correlations were within-team centered variables.

As a first step, we estimated the amount of variability due to intra-team differences by examining the intraclass correlation coefficient type 1 (ICC1; Bliese, 2000), which indicates the amount of variance in the dependent variable that results from between-teams’ differences. The ICC1 indicated that between-person variance explained 20.91% of the variance in perceptions of team adaptive performance (event-based adaptive performance). These findings show that our multilevel analytical approach is appropriate, as we controlled for events nested in teams (Bliese, 2000). We next present our results.

In Model 1 (see Table 3) we entered the three team adaptation processes as predicting perceptions of team adaptive performance in the same model, that is, simultaneously (with all three processes jointly competing for team adaptive performance variance). The results reported in Table 3 suggest that adaptation of interpersonal processes (*Estimate* = 0.41, SD = 0.11, t=3.66, *p* < .000) and transition processes (*Estimate* = 0.22, SD = 0.10, t = 2.24, *p* = .029) were positively related to perceptions of team adaptive performance, thus supporting *H1b* and *H1c*. Differently, *H1a* was rejected because the adaptation of action processes was not significantly related to perceptions of team adaptive performance (*Estimate* = 0.10, SD = 0.12, t = 0.84, *p* = .401).

**Table 3.** Results of Hierarchical Linear Model Analysis to Test Hypothesis 1 and 2.

	Model 1 (h1)		Model 2a (h2a) – Action processes		Model 2b (h2b) – Transition processes		Model 2c (h2c) – Interpersonal processes	
	Coeff. (SD)	*t p*-value	Coeff. (SD)	*t p*-value	Coeff. (SD)	*t p*-value	Coeff. (SD)	*t p*-value
Intercept	1.40 ** (.40)	3.46 .001	1.93 * (.79)	2.43 .018	1.73 * (.85)	2.05 .045	1.88 * (.81)	2.31 .025
Team adaptation processes								
Action processes adaptation	0.10 (.12)	0.84 .401	0.03 (.24)	0.14 .887	0.08 (.13)	0.55 .582	0.07 (.13)	0.60 .546
Transition processes adaptation	0.22* (.10)	2.37 .029	0.24* (.11)	2.19 .032	0.2 (.24)	1.02 .315	0.24* (.11)	2.19 .033
Interpersonal processes adaptation	0.41** (.11)	3.66 .001	0.43** (.12)	3.64 .000	0.42** (.12)	3.68 .000	0.40 (.12)	1.53 .130
Event characteristics								
Event criticality			−0.19* (.32)	−0.61 .54	−0.11 (.36)	−0.31 .757	−0.18 (.33)	−0.53 .596
Interactions between team processes and trigger characteristics								
Event criticality * action processes adaptation			0.01 (.07)	0.18 .855				
Event criticality * transition processes adaptation					−0.00 (.08)	0.07 .946		
Event criticality * interpersonal processes adaptation							0.00 (.08)	0.13 .899
Additional information of model estimation								
AIC	229.05		237.40		237.15		237.40	
BIC	243.71		256.76		256.50		256.75	
Loglik	−108.53		−110.70		−110.58		−110.70	

*Note.* Hypotheses testing was performed at the event level of analysis (*N* = 86 events). Team performance was the dependent variable. Asterisks in the first column signal interactions between variables. Asterisks in all remaining columns signal statistically significant relations (*p* values are presented in the table). *p < .05; **p < .0.

Regarding *H2* (Model 2), we tested the moderation effect of event trigger criticality on the relationship between action (*H2a*), transition (*H2b*) and interpersonal process (*H2c*) adaptation and perceptions of team adaptive performance. The results reported in Table 3 show that we did not find any empirical support for those 3 hypotheses, as no interaction effect was significant. Respectively, for *H2a: Estimate = 0.01, SD = 0.07, t = 0.18, p = .855; for H2b: Estimate = −0.00, SD = 0.08, t = 0.18, p = .855; and for H2c: Estimate = 0.00, SD = 0.08, t = 0.13, p = .899)*. This shows that, according to our data, the impact of team adaptation processes on perceptions of team adaptive performance did not change based on event trigger criticality.

Regarding H3 (Model 3), we studied how event trigger criticality and event trigger origin jointly moderate the relationship between team adaptation processes and perceptions of team adaptive performance. As detailed in Table 4, we tested separate models of each team adaptation process and the two event trigger characteristics. We found a significant three-way interaction for action processes adaptation (*Estimate* = 0.47, *SD* = 0.14, *t* = 3.23, *p* = .002), transition processes adaptation (Estimate interaction = 0.55, *SD* = 0.17, *t* = 3.15, *p* = .003), and interpersonal processes (*Estimate* = 0.56, *SD* = 0.15, *t* = 3.62, *p* < .000). These findings mean that the relationship between team adaptation processes and perceptions of team adaptive performance changed at different levels of event criticality and event origin combined. In other words, the way that event criticality changed the relationship between adaptation processes and perceptions of team adaptive performance depended on event origin. We elaborate on these findings in the following section.

**Table 4.** Results of Hierarchical Linear Model Analysis to Test Hypothesis 3.

	Model 3a (h3a) – Action processes		Model 3b (h3b) – Transition processes		Model 3c (h3c) – Interpersonal processes	
	Coeff. (SD)	*t p*-value	Coeff. (SD)	*t p*-value	Coeff. (SD)	*t p*-value
Intercept	−6.98 * (2.32)	−2.99 004	−7.85 * (2.59)	−3.02 .004	−9.11** (2.49)	−3.64 .000
Team adaptation processes						
Action processes adaptation	2.13** (.61)	3.50 .001	0.01** (.13)	0.12 .905	0.07 (.12)	0.60 .549
Transition processes adaptation	0.24* (.11)	2.18 .034	2.63** (.67)	3.91 .000	0.18 (.11)	1.75 .086
Interpersonal processes adaptation	0.39** (.12)	3.32 .001	0.39** (.11)	3.29 .002	2.96** (.62)	4.74 .000
Event characteristics						
Event origin	6.78** (1.61)	4.20 .000	6.78** (1.61)	6.82 .000	7.99** (1.71)	4.65 .000
Event criticality	2.39** (0.82)	2.90 .005	3.24** (1.05)	3.07 .003	3.19** (0.92)	3.44 .001
Two-way interactions						
Event origin * team processes adaptation	−1.58** (0.40)	−3.96 000	−1.64** (0.44)	−3.72 .000	−1.84** (0.40)	−4.52 000
Event criticality * team processes adaptation	−0.59** (0.20)	−2.85.007	−0.81** (0.25)	−3.18 .002	−0.76** (0.21)	−3.48 001
Event origin * event criticality	−2.02** (0.60)	−3.43 001	−2.32** (0.72)	−3.20 .003	−2.43** (0.65)	−3.73 .000
Three-way interactions						
Event origin * event criticality * team processes adaptation	0.47** (0.14)	3.23 .002	0.55** (0.17)	3.15 .003	0.56** (0.15)	3.62 .000
Additional information of model estimation						
AIC	221.15		222.27		216.12	
BIC	248.47		249.59		243.44	
Loglik	−98.57		−99.13		−96.07	

*Note.* Hypotheses testing was performed at the event level of analysis (*N* = 86 events). Team performance was the dependent variable. Event origin was coded as “team” (1) and “environment” (2). Asterisks in the first column signal interactions between variables. Asterisks in all remaining columns signal statistically significant relations (*p* values are presented in the table). *p < .05; **p < .01.

#### Discussion Study 1

In Study 1, we aimed to test if the relationship between team adaptation processes and perceived adaptive performance was moderated by the criticality and the origin of events. Overall, the findings suggest that when team adaptation processes alone are considered, the adaptation of transition and interpersonal processes has more to add to team adaptive performance than the adaptation of action processes. However, when the context of adaptation is regarded by considering the characteristics of event triggers, all team adaptation processes contribute to team adaptive performance to some extent. Specifically, our findings suggest that the perception of event criticality is influenced by whether the event occurs within the team or the environment. This distinction, in turn, affects the degree to which team adaptation processes impact perceptions of team adaptive performance. When the event trigger emerges from the environment, modifying all three types of team processes is positively related to perceptions of adaptive performance when the trigger is also highly critical. This diverse team response may arise due to a sense of urgency that motivates individuals and teams to act, given the negative consequences of inaction (e.g., evading a floating iceberg or animal attacks). In contrast, with triggers of low criticality, modifying all three types of team processes is negatively related to perceptions of adaptive performance. This finding may be situation-dependent. For example, when dense fog prevents flight operations or the collection of satellite or aerial imagery, the only viable option is to wait and postpone operations until conditions improve (e.g., *not* adapt anything).

**Figure 2.**
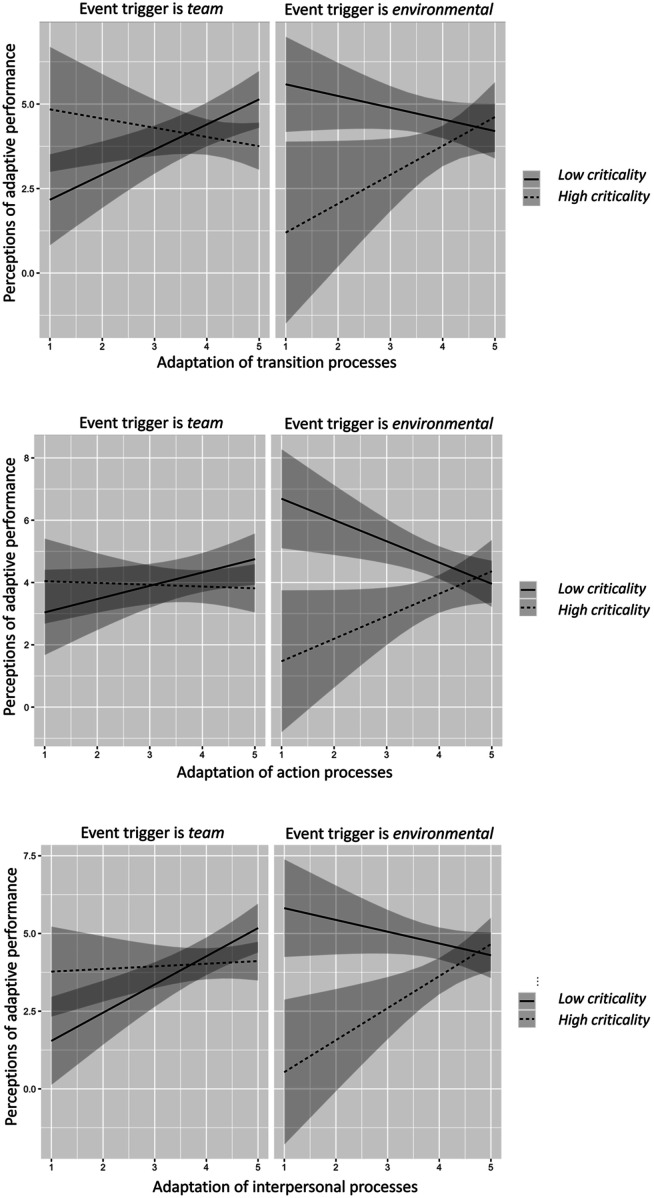
Predicted values of team adaptive performance as function of action processes, transition processes, interpersonal processes, event trigger criticality and event trigger origin.

As detailed in Figure 2, the relationship between team adaptation processes and team adaptive performance is different for team-based triggers. Namely, the modification of the three types of team processes is positively related to perceptions of adaptive performance only when the trigger is also of low criticality (see Table 4 and Figure 2). This suggests that for team-based triggers, modifying team processes leads to enhanced perceptions of adaptive performance only for events that can be easily resolved. For instance, intense conflict within or between teams may result in avoidance or withdrawal behaviors. Given the short duration of the mission, individuals may recognize that engaging in confrontational or conflict management behaviors is unproductive (Larson et al., 2019; Palinkas & Suedfeld, 2021).

Through the findings in Study 1, we have obtained initial evidence of the nuanced relationship between team adaptation processes, perceived adaptive performance, and the characteristics of the events that trigger team adaptation. However, in this study, other dimensions of event characteristics were not considered, which could also play a significant role in team adaptation in extreme environments. Indeed, the nuanced relationships between event characteristics, team adaptation processes, and perceptions of team adaptive performance that Study 1 revealed call for an in-depth analysis of the events teams’ face and how they adapt to those events. This motivated conducting a follow-up study, as we explain in the following section.

## Method - Qualitative Study 2

We undertook a complementary and comprehensive qualitative study to enhance our insights derived from the quantitative investigation in Study 1. This qualitative approach aimed to add richness to the events and adaptation processes transpiring within extreme environments as experienced first-hand by research participants and to broaden the scope of our research model (see Figure 1). In so doing, our intent was to provide contextual depth to the outcomes gleaned from our quantitative analysis and facilitate a richer understanding of team adaptation under different event characteristics. This crucial second study allows us to explore the variability and complexity of adaptive processes, providing a more holistic view of how teams respond to diverse and unpredictable triggers. Furthermore, the qualitative insights can help identify potential gaps or limitations in the initial quantitative findings, thereby offering a more robust and comprehensive understanding of the dynamics at play in extreme environments.

We conducted in-person interviews with a diverse array of scientists and staff stationed across various research stations in Antarctica. These immersive interactions afforded us a nuanced perspective on the intricacies of the events under scrutiny. Additionally, we expanded our purview by examining distinct facets of event triggers, encapsulating characteristics that contribute to event robustness, namely novelty, criticality, and disruption, as delineated by the EST (Morgeson et al., 2015). Our qualitative investigation incorporates elements of novelty and disruption, augmenting our quantitative work that only focused on the criticality dimension of event strength. By doing so, we seek to refine our evaluation of event potency, deepening our understanding of the repercussions stemming from events originating from diverse sources. This approach thereby facilitates a more nuanced dissection of the impacts induced by distinct event origins upon team adaptation dynamics.

### Sample and Procedure

As the second component of the sequential explanatory mixed methods approach, we extracted data from participant semi-structured interviews conducted as part of a larger 3-year study of Antarctica teams, focused on the drivers of effective teamwork in extreme environments (for the full interview script, please see the Appendix). In the current study, we included only the data (*N*=20) from one year (2020) – the same year the quantitative study was conducted. The interview transcripts were completely reanalyzed with a new focus on events, and none of the exemplary quotations included in this study have been published previously.

We applied a purposive sampling strategy to recruit team members with the same backgrounds as our quantitative sample. Participants represented all roles (N_Staff_ = 8, N_Scientists_ = 10, N_Builder_ = 1, N_Station manager_ = 1), a variety of nationalities (N_Chile_ = 7, N_Uruguay_ = 5, N_Portugal_ = 5, N_Germany_ = 1, N_Ecuador_ = 1, N_United Kingdom_ = 1), and were staying at different stations representing different organizational cultures (N_Profesor Julio Escudero Base_ = 10, N_Artigas Base_ = 6, N_Juan Carlos I Base_ = 2, N_Antarctic Great Wall Station_ = 1, N_Belinghausen Station_ = 1). Nine participants remained in Antarctica for the complete summer season (October-March) and 11 stayed between 2 weeks and 2 months depending on the project they were working on. Eight participants were female and 12 were male. Since the interviews were conducted during the same period and at the same research stations where the quantitative study was conducted, all 20 interviewees also participated at some point, independent from the interviews, in the quantitative study.

The interviews were conducted in English, Spanish, or Portuguese. The first and second authors conducted the interviews together. Either the first or the second author took the lead while the other took notes and asked additional probing questions. Interviews were recorded and transcribed for further analysis and lasted between 45 and 90 minutes. Every participant provided explicit verbal consent. Since the interviews were part of a larger, more general project about teamwork in Antarctica, the interviews explored a variety of aspects including team composition, experience, training, interpersonal relationships, and critical incidents. For this study we focused mainly on the portion of the larger data set that focused on the description of critical incidents (i.e., events) and reactions to them which we probed via questions about the experience of unexpected events or challenges in Antarctica.

### Analytical Approach

**Figure 3.**
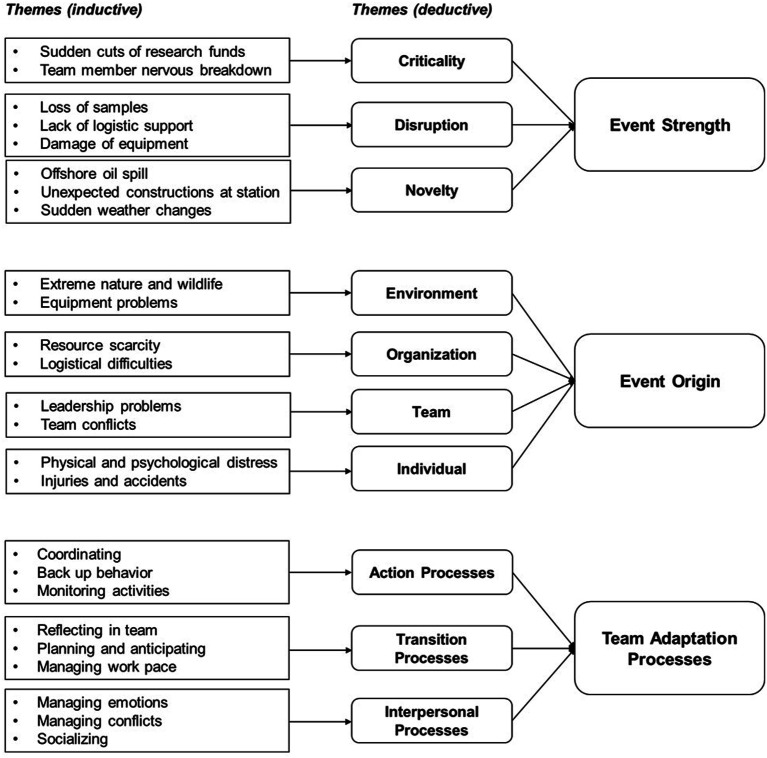
Qualitative data structure and the themes that emerged from the content analysis, for event origin and team processes.

For the qualitative study, we focused our analysis on events reported by individual team members and how their teams managed these situations. These events included situations that occurred during the mission in 2020, when the data was collected, and occurrences that took place in previous campaigns that we considered relevant enough to include in our analysis. We applied a deductive and inductive approach to analyze the qualitative data. First, we applied a priori framework to the data (Goldsmith, 2021) using two underlying theories from the quantitative study: the EST (Morgeson et al., 2015) and the team processes adaptation framework (Maynard et al., 2015). We systematically applied the frameworks to the data and indexed events and labeled their origin according to the four levels (organization, environment, team, and individuals) as well as the event effect (i.e., bottom-up, top-down, same-level); and their strength. We did the same for the three team processes: action, transition and interpersonal. During the deductive stage of data coding, we applied existing themes to our data for a general characterization of event characteristics and team adaptation processes. After the first stage, our coding focused only on the indexed segments (i.e., events and team processes) and our analysis identified themes using an inductive approach. These themes were pulled directly from the data and fitted within higher order themes as shown in Figure 3. The first and second authors reviewed the initially identified segments and applied open codes to classify the segments. These two authors engaged in multiple rounds of coding, re-reading, note-taking, and discussion to resolve disagreements are develop shared understanding about the codes assigned to segments before proceeding with summarizing codes into higher order themes (Clarke et al., 2015).

### Results

The results section is organized in two parts. First, we focus on further examination of our quantitative findings by searching for evidence in our interview data that could complement the nuanced relationship between team adaptation processes and perceptions of team adaptive performance, given the characteristics of adaptation triggers. Second, we focus on expanding the scope of our findings by looking at team adaptation under additional event characteristics (see Figure 1).

Before proceeding, we highlight the data structure and themes from the general qualitative analysis (Figure 2). Using the EST (Morgeson et al., 2015) as a guiding framework, the deductive approach revealed examples of all dimensions of event strength (i.e., criticality, disruption, and novelty) and origin (i.e., organization, environment, team, and individual) dimensions. The deductive approach also allowed us to identify the team adaptation processes that were enacted when teams dealt with different events. Additionally, the deductive approach also revealed relevant examples of adaptation of the three team process categories: action, transition and interpersonal (Maynard et al., 2015). Finally, codes for event origin and team processes were also derived inductively to gain better understanding of specific challenges teams faced, and the behaviors in which they engaged to deal with such events (see Figure 2). Further analysis of the categories of event strength, event origin and team processes revealed several context specific themes (presented in italics) that are summarized in the following.

### Further Examination of Quantitative Findings

In Study 1, we found that the relationship between team adaptation processes and perceptions of team adaptive performance was stronger when teams faced events in their *environment* that had *high criticality*, as well as when they faced events in the *team* that had low criticality. In line with EST (Morgeson et al., 2015), we identified events in the qualitative analysis as highly critical if they either threatened life (e.g., injury or death) or led to/or were a risk that could lead to the cancellation of the entire mission. Conversely, we considered events as *low criticality* if they resulted only in delays to the mission or led to challenges that could be resolved within a reasonable time frame. This classification was based on the potential impact and severity of the event’s consequences on the mission’s overall success and the safety of the participants (Morgeson et al., 2015). Furthermore, we determined event origin based on where the event originated, including ‘environment’ and ‘team’. In our data, the environment represented everything outside the organization, including Antarctica’s harsh nature and wildlife, whereas the team represented everything happening within the team or that was somehow directly related to teamwork and taskwork.

#### Environment - High Criticality

For example, one event that originated in the *environment* and had *high criticality* was the sudden reduction of research funds by governmental agencies (environment – high criticality). Surprise cuts in funding had high criticality in terms of conducting scientific activities because general resources were scarce and opportunities to buy or borrow equipment were few. Surprise cuts had a top-down effect on research teams’ capacity to do their work. When this occurred, many individuals returned home with no data and only a few adapted to the situation and completed their projects:

“The resources did not arrive, the money did not reach from the agency that provides the money to buy the equipment, we did not have equipment, we only had very simple equipment that are quadrants that help measure abundance of species (…) there were colleagues who had not been able to do anything, absolutely nothing and have had to return to the continent without data, without anything.” (P10).

Project completion was achieved through transition processes in which team members adapted to collectively reflect and reevaluate their options, and if it was feasible to continue the mission or cancel. This required new planning, a realistic reassessment of resources and mission objectives, and the adjustment of work pace:

“The original objective was to have measurements of solar radiation for algae (…) which required this more sophisticated equipment. Since it didn't come, I thought, “Well, maybe we can just make simpler estimates,” which requires just the eyes and an estimate of how abundant these species are, we can do that.” (P10).

Teams also adapted through action processes by providing support to each other (including sharing resources) and coordinating amongst themselves and logistic personnel to improvise new methods and tools with eventual success:

“We improvised something, a small study to measure the abundance of algae and animals around the island.” (P10).

#### Team - Low Criticality

As for events in the team that had low criticality in the context of the Study 1 findings, some examples include one participant reporting that the team had to grow vegetables using wastewater in an improvised device because the specialized device they were bringing was lost on the way to Antarctica. One participant also lost a special device to get water samples from the sea but managed to build a new device using cans and bottles that they found at the research station. This required asking for support and improvisation (action processes), as well as readjusting the mission schedule given the loss of time (transition processes):

“Here, devices tend to work less, batteries tend to work less, things tend to break because of the weather, so you can be unlucky, and you have to understand that (…) a lot of things brake, a lot of things (…) Somehow, they get solved (…) We didn't have the things to tie some bags to the bottom of the lake, and we asked the scuba divers here, and they made it for us (…) they worked even better than you would have done it yourself probably, so asking for help is the answer. Ingenuity, and asking for help.” (P4).

The reported events related to equipment problems and extreme nature and wildlife mainly had a top-down effect on the team. These events impacted the research mission by delaying all work or portions of the work that could not be carried out when important equipment was missing. To adapt to these circumstances, teams relied on their social network within research stations built through interpersonal processes, to engage in action processes like looking for solutions that could either help them retrieve their equipment on time or find the necessary resources to build an improvised tool that could satisfy their needs. In situations like these, teams also had to engage in transition processes by rethinking their mission plan, which included readjusting mission goals and research protocols.

In Study 1 we also found that the relationship between team adaptation processes and perceptions of team adaptive performance was weaker when *teams* faced events related with the *environment* that had *low criticality*, and events that emerged from within the team that had *high criticality*.

#### Environment - Low Criticality

For example, the former case refers to dealing with weather changes that prevented field work activities to be performed, either because they disrupted data collection, created unsafe conditions, or a combination of both. In these cases, the only thing researchers could do was to accept the fact that field work was not possible and so they had to wait patiently. As an adaptation processes, team members could only engage in transition processes such as reflecting about their missions’ goals and plans, to decide the extent to which such delay could impact mission success and what could be done to mitigate that impact. In these situations, teams could also engage in interpersonal processes to preserve morale and stay focused despite the monotony (and sometimes anxiety) of the wait. Apart from weather changes, one team encountered an elephant seal one night that crossed their camping site and knocked down several tents and equipment. Once the seal went its way, the only solution was to set camp again. Once the initial surprise of the encounter passed, teams engaged in action processes by helping each other reorganizing camp and get back to sleep:

“We had to fix it, but this happened in the middle of the night, toward the end of February, so everything was dark. We managed to scare the animal away and then rebuild our tent (…) the next day we took it with good humor (…) we managed to rebuild the camp too and finish the campaign without a problem.” (P2).

#### Team - High Criticality

Finally, one example of an event that originated in the *team* and which had *high criticality* was that sometimes team members became physically and mentally exhausted and were unable to continue working efficiently and safely. During one short-duration mission the team leader’s priority was to conduct and complete the research project. On the one hand, leaders sometimes pushed their team members to complete high workloads. On the other hand, less experienced team members sometimes saw the expedition more like an adventure with many opportunities to collect extra data, have fun, or in some cases even get romantically involved with other researchers. All of these actions served to distract from the main purpose of the Antarctica stay:

“Some relationships occur sometimes. Everything is fine until the group is not affected (…) some stress problems. (…) I had some frustration because she acted a bit like the princess here (…) also in other bases, they have one or two women and then everything is focused on you. Some feel like, “I'm the most beautiful. I'm the best (…) Sometimes, it's not nice for the rest of the team looking at this and it’s like, “What's going on?” It's the small things.” (P16).

Thus, such events could potentially hamper the research mission. To deal with these situations, team members had to speak up, pause work for a day or two, or reduce work pace. In both cases, there was little that could be done in terms of adapting team processes to solve the situation, which helps explain the weaker connection between team adaptation processes and perceptions of team adaptive performance in these conditions.

Similarly, another example was a nervous breakdown by a fellow team member who realized that did not want to be in Antarctica, away from family, only after arriving to the research station and with no possibility of going home before the mission ended:

“As we arrived, the first day she flips, she arrived to the room and she started like punching the walls and saying, “You can't talk to anyone if I'm not talking.” (…) When I got confronted with that, (…) it was really a struggle (…) Then I realized that she didn't want to be here. She wanted to do the work, but she didn't want to be far away from her family and from her boyfriend” (P19).

This situation, which could also qualify as a disruptive event, led to a dramatic deterioration of the team social structure, with the team member self-isolating and not being able to contribute much to the mission implementation nor the team climate. Left alone, the other team member had to look for minimum support from the station management and logistics, to do as little work as could be done. However, the team was unable to conveniently adapt its processes and therefore their ability to perform adaptively was poor:

“We stayed for 20 days, and she was tired, and I wouldn't push because I was always afraid of her reaction, (…) the staff was very nice, so we talked, and everybody would start talking to her. She would then leave and didn't say anything to any of them, but then she would yell at me in the bedroom “ you talk too much, why are you talking to them? I don't need to talk if they want to talk with you!” (…) you don't want to be in a place where someone from your team creates a conflict with everybody, and she did in the last day with the boss at the station, but they are really nice, so nothing happened. For me, that's the worst part.” (P19).

### Team Adaptation under Additional Event Characteristics

Up to this point, we have presented a summary of the qualitative research findings that help illustrate what was found from the quantitative study. In the following section, we expand on these findings by looking at the relationship between additional event characteristics and team adaptation. In line with EST (Morgeson et al., 2015), we looked beyond criticality to expand our understanding of team adaptation in extreme environments by also considering event disruption and novelty (event strength), as well as additional levels of event origin (individual and organization).

### Event Strength

In event strength, we looked for event disruptions by searching for evidence in our data that spoke to clear discontinuities in operations that led to changes in the conditions under which teams were performing. We also looked for event novelties, reflected in events that were somehow unusual or described as different from current and past situations.

#### Disruption

Events in ICE environments such as Antarctica could also be disruptive. For instance, scientists working with living samples such as fish, crustaceous or algae might lose their samples and were unable to understand why, or what caused it. Events such as this had a single level effect because their impact remained within the team and led team members to experiment with alternative research protocols through transition processes like *planning and anticipating* or *reflecting in the team*. Other disruptive situations included being denied access to logistic support due to the lack of sufficient resources and the need to prioritize projects, or having critical equipment damaged with no chance of repairing:

“It happened last season that our boat broke and, coordinating with Chile, they made trips to the sea for us. That happens a lot, it requires coordination from base leader to base leader, but that's how it is done” (P6).

Our findings suggest that in situations such as these, when events had a top-down effect on teams and individuals, teams benefitted from engaging in action processes and interpersonal processes such as *back-up behavior* by asking for help from other research stations or developing good interpersonal relationships and broadening one’s network through *socializing*. Also, research stations closely *monitored the performance* of each team so that they were able to engage in *back-up behavior* and provided help if needed (an example of action processes).

#### Novelty

There were a few unique situations that could also be regarded as novel, including an oil spill offshore, and having construction workers around:

“At the beginning it was complicated because they did not understand the concept of research or a scientist. I had to be constantly instructing, telling them that there were people working, there are researchers who need silence - They understood as time went by, (…) they have been here for three months.” (P12).

These events prompted scientists and station managers to engage in transition processes such as *reflecting in the team* to find a solution to accommodate them. However, scientists also dealt with Antarctic politics, which made solving the situation more difficult and aggravated the top-down influence of new events on the team. Likewise, both transition processes such as *reflecting in the team*, or interpersonal processes such as *managing conflict* between teams were an important part of the adaptation process to events such as the arrival of a construction crew to a research station, who were often unfamiliar with station *etiquette* rules such as putting on station shoes upon entry and keeping noise levels down when other people were either working or resting. Situations like this were unusual, and team leaders played an important role in *managing conflict* and emotions between subgroups (e.g., scientists vs. builders):

“We have been talking between researchers and us to understand that this also has to happen because while you are working you cannot tell the builder not to hammer if he has to hammer(…) Both parties have done their part to make it work (…) there is one person who was more interested in asking what they were doing, why they were here (…) there was someone who was asking, “What are they doing? Why do they do it? Why do they read? Why do they filter water or why do they do certain things,” so it was very interesting.” (P12).

### Event Origin

In Study 1, we had already looked at events that originated in the environment and in the team. In Study 2, we aimed to deepen our understanding or the role of event origin by also searching for evidence in our data that reflected organizational and individual originated events. The organizational level in this dataset represents the research station at which a team is based. Each research station housed several teams and individuals for which the station management was responsible. The research station as an organization was tasked with providing the necessary resources to the research teams (e.g., logistical support, basic equipment, food, lab space) and individuals. The individual level in this dataset represents the individual team members that were part of the different teams that populate the Antarctic environment during the summer months, and which were part of our sample.

#### Organization

We considered the base station to be at the organizational level, which was also the origin of some significant events. Events were reported related to *limited resources* at the station. One participant reported that food supplies were running low, the variety and abundance of food available at the research station decreased dramatically. The cooks had to change their meal plans (transition processes) to keep the crew’s morale high (interpersonal processes), including rationing supplies and improvising new meal courses by reorganizing food ingredients in different ways (action processes). This is highlighted by the following quote:

“Two weeks ago, we started running out of food supplies. When the food starts to run out you must start improvising and get people to accept that. Maybe it's a little difficult, because sometimes people don't understand that every day you get French fries and chicken. They're going to say, “Don't the chefs know how to make something else? What is this?” (P11).

Other events were related to *logistical difficulties* at the base station. For example, participants reported some events in which the research station could not provide logistical support such as a boat that was necessary to get water samples at sea: *“This year, the station chief was obviously not willing to help with the Zodiac transport (…) and they had problems with the engine”* (P16). To solve this problem, researchers had to look around and use their network to find support and arrange alternative transport (action processes): *“It’s also that you have good connection to the others, and you can ask for help”* (P16). Limited resources and logistical difficulties had a top-down effect on teams as well as individuals as they disrupted operations and forced team members to change strategies (transition processes) and look for support elsewhere (action processes).

#### Individual

At the individual level, participants reported events related to *physical and psychological distress and injuries and accidents*. If researchers or staff stayed in Antarctica for several months, the isolation from friends and families led to feelings of loneliness or even depression in the worst cases:

“The power generation was only for two hours at night. Therefore, everything was very precarious. However, when we arrived there (…) one of them started to feel depressed (…) at some point, he broke down and said that he’d started missing daily things again, because he hadn’t talked with anyone, but now that he spoke with us and let it all out (…) Therefore, I say it was a very special and friendly group.” (P2).

This account speaks to the importance of interpersonal processes like *emotional support* to help individual team members while also keeping the team morale high.

Besides psychological distress, work in the harsh environment was physically exhausting. Sometimes researchers could only stay in Antarctica for one or two weeks. Therefore, they needed to maximize the use of this time for data collection. This included long hikes to remote places and long working hours outside under cold and windy conditions that were physically exhausting:

“We wake up early. We work the whole day, like 12–15 hours sometimes, so the biggest challenge for me is physical because it's very physical. Even if you have a day in the lab, it's still very physical. You must do a lot of things and worry about a lot of things, so that is a challenge for me, at least (…) You always think like, “Okay. If I don't do it today, maybe I have the next five days are going to be awful and I put a foot out the door. I'm going to lose a lot of time.” (P4).

How teams balance this is by prioritizing work and rearranging work routines to always be in pairs (transition processes), adjust work pace, and ask for additional help from others if the available number of people is not enough (action processes).

The reported events originating from the individual level had either a single-level effect or a bottom-up effect on the team. Injuries, accidents, and individual distress in general affected individual well-being and may eventually negatively affect an individual’s ability to conduct work. In the most extreme case, team members could not carry out further work, threatening mission success. Team member well-being had the highest priority. Therefore, team members redirected their resources towards an individual that experienced a negative event, and the mission became secondary. Overall, the teams in our dataset were confronted with numerous events amid extreme conditions and isolation, necessitating significant adaptation. These challenges, ranging from funding cuts to equipment failures, disrupted research endeavors and required quick adjustments to team processes. The event origin, whether environmental or organizational, further complicated matters, impacting team dynamics and mission fulfillment. Despite these obstacles, teams adapted by building bonds of camaraderie (interpersonal processes) and a shared pursuit of knowledge that helped prioritize mission goals and the well-being of individual members (transition processes) and executing their work plan (action processes).

## Discussion

Our research investigated team adaptation in extreme environments. Our goal was to examine how event trigger characteristics influenced the relationship between team adaptation processes and perceptions of team adaptive performance. We did so by integrating literature from team adaptation (e.g., Maynard et al., 2015) and extreme environments (e.g., Golden et al., 2018) within the EST framework (Morgeson et al., 2015), and then conducting two field studies in Antarctica to collect and analyze data following a mixed-methods, event-based approach. Our findings highlight that the modification of transition and interpersonal processes had positive relationships with perceptions of team adaptive performance with modification of interpersonal processes holding the strongest relationship. This is an important finding given that research investigating the relationship between processes and adaptive performance has not examined interpersonal processes extensively and certainly has not examined the relative salience of each of the Marks et al. (2001) team process categories within a single study which allows for a comparison of their salience in shaping perceptions of team adaptive performance.

The outcomes of this research also reveal boundary conditions for the team adaptation processes – team adaptive performance relationship, based on the strength and origin of the trigger being faced by the team. Specifically, our findings suggest that when a team faces a trigger emerging from the broader environment, the extent of modifying team processes has different effects on perceptions of team adaptative performance depending on whether the trigger is of low or high criticality. Our results suggest that when teams face an environmental trigger of low criticality, they are best off not modifying any of their processes significantly. In fact, if teams adapt action, transition, and interpersonal processes primarily when facing environmental/low criticality triggers, perceptions of team adaptive performance decline. It may be that with triggers that emerge from outside the team’s control and are not that critical, less is more in terms of altering team processes. In contrast, for more critical environmental triggers, our results suggest that failing to modify each team process type is detrimental to perceptions of team adaptive performance. In such situations, the team’s performance is enhanced by modifying each team process type.

When looking at triggers that emerge from within the team, the results are quite different. To be more precise, for team-based triggers that are low criticality, failing to modify team processes has detrimental effects on perceptions of team adaptive performance. In such situations, the team is better off by modifying each type of team processes. In comparison, when a team faces a highly critical team-based trigger, the team needs to respond in a more nuanced fashion. Specifically, as noted in Figure 2, in such situations altering the action processes seems to have negligible effects while modifying the transition processes seems to have a small negative effect on perceptions of team adaptive performance. For these team-based/highly critical triggers, the team is best off by modifying interpersonal processes. This is, in part, intuitive as the trigger emerges from within the team and therefore, the team needs to be cognizant of the impacts on the established team member relationships. These relationships deserve more attention in future research and speak to our contribution that emphasizes the need for teams to also consider interpersonal processes when facing triggers in extreme contexts.

To summarize, our findings suggest that the relationship between team adaptation processes and perceptions of team adaptive performance was more positive under two conditions: (a) when teams faced event triggers that originated from the team and had lower criticality; and (b) when teams faced event triggers that originated from the environment and had higher criticality.

The outcomes of the qualitative study build upon the findings of our quantitative study. To start, the qualitative study provided details of the types of triggers experienced by individuals and teams in Antarctica and how they can be categorized in terms of EST dimensions of criticality, disruption, and novelty. Additionally, our interviews provided more in-depth understanding of trigger characteristics in terms of their origin from either environment, organization, team, or individual. Finally, the interviews shed more light on their responses to triggers in terms of necessary adjustments to action, transition, and interpersonal processes. For example, we learned more about the importance of interpersonal processes within extreme environments. Namely, interpersonal processes and modifying them in the face of triggers seem particularly salient in such situations as they help teams keep working together despite the lack of privacy and forced interactions. Also, when teams are cut off from management support, they rely on their interpersonal relationships to find other ways to get the resources they need to complete their missions. These findings align with prior research (e.g., Burke et al., 2018), as well as with historical evidence (e.g., Shackleton, 2015) about teamwork in extreme environments. Finally, a nuanced understanding of extreme environments requires a deep immersion within specific contexts; specific requirements can be hardly gleaned from research with typical organizational teams (Maynard et al., 2021). The rich insights from our qualitative data about events and team processes show the potential for future studies along these lines. To clarify the effectiveness of different adaptation processes, future work needs to emphasize the characteristics of the different contexts in which the teams work, and deliberately investigate team adaptation under varying degrees of extremeness (Schmutz et al., 2023).

### Theoretical Implications

Our research bridges the taxonomy of team processes (Marks et al., 2001), the team adaptation literature (e.g., Maynard et al., 2015), and the extreme environments literature (e.g., Schmutz et al., 2023), while also integrating concepts from the EST framework (Morgeson et al., 2015). To do so, we have addressed recent calls to combine quantitative and qualitative methods to study the interplay between adaptation triggers (as events) and team adaptation in extreme environments (e.g., Maynard et al., 2021). Also, the focus on extreme events enables us to apply our findings to more traditional teams because most teams in today’s organizations eventually face events that demand adaptation (Hällgren et al., 2018). Moreover, by combining quantitative and qualitative methods to study teamwork in extreme environments, we could disentangle the importance of action, transition, and interpersonal processes for team adaptation.

Finally, despite taxonomies of adaptation triggers differing to some extent across studies, there is consensus that the characteristics of the event triggers for adaptation play a role in determining under which conditions team adaptation may lead to enhanced team performance (e.g., Georganta et al., 2019; Maynard et al., 2015). In this study, we have investigated event trigger characteristics in an integrative way by considering the role of multiple event trigger characteristics (e.g., criticality and origin) as they interact with each other to shape the relative salience of modifying different types of team processes in leading to enhanced perceptions of team adaptive performance. In so doing, we have helped provide empirical evidence to EST (Morgeson et al., 2015) and extended the work of Maynard et al. (2015) and Christian et al. (2017) by examining how event characteristics shape team adaptation dynamics.

### Practical Implications

Our findings provide insight into the potential of training teams to anticipate and identify cues that signal the need to engage in process adaptation to ensure effective performance in extreme environments and beyond. Through the creation of management routines that help team members engage in team adaptation processes during missions in extreme environments, teams in such contexts and others are more likely to develop effective strategies to ensure good performance (Landon et al., 2018). Given our results, managers and team leaders should emphasize opportunities for their teams to adapt their transition and interpersonal processes as these seem to be the most salient within extreme contexts. This can be achieved by creating daily opportunities for individuals to engage in interpersonal processes (i.e., during mealtimes or social events) and transition processes (i.e., during group meetings where tasks, roles and responsibilities are defined).

What is more, our findings also inform human activities in extreme environments besides Antarctica, including human space flight where academic and practitioner communities are profoundly interested in learning more about factors that develop, maintain, and restore group affective states such as cohesion. By expanding on previous evidence that shows how team interpersonal processes contribute to team cohesion and team trust (Golden et al., 2018; Palinkas & Suedfeld, 2021), our findings show that team interpersonal processes greatly contribute to team performance in extreme settings when adaptation is important and therefore are more critical to be developed and maintained prior to as well as during missions.

### Research Limitations and Future Directions

Field research involving teamwork in extreme environments is rife with numerous challenges, including the difficulty in accessing study participants, gathering *enough* data to perform sound statistical analysis, and the remoteness of data collection sites. Such challenges often lead field researchers to make compromises between methodological rigor and the operational constraints of the research environment (Bell et al., 2018; Landon et al., 2018). While we feel we creatively overcame many of these challenges with an event-based and mixed-method approach, our current research also has limitations. According to our goals, we focused on events, modifications of team processes, and team members’ perceptions of team adaptive performance. As a result, our data was collected at one point in time (by reconstructing team members’ perceptions of their team’s experiences in a single event). While such a research design is cross-sectional, it is appropriate in cases where researchers conduct empirical studies for which there is little work on which the research hypotheses can be solidly grounded and in cases where there is limited access to bigger samples (Spector, 2019).

Another limitation relates to sample composition, namely, the diversity of nationalities and the gender balance in the study sample. Although the research team used native speakers to translate the surveys and all psychological measures had been previously validated (e.g., Mathieu et al., 2020), this study still lacks culture-specific duly validated questionnaires or the consideration of cross-cultural dynamics (Landon et al., 2018). Despite the adequate reliability of the adopted questionnaires, our small sample size prevented us from performing more complex psychometric comparisons and additional analysis. As such, we call for future research to continue to explore and provide evidence for the cross-culture validation of the measures utilized within this study. What is more, as other studies have examined all-female teams (Kahn & Leon, 1994), therefore future research may want to examine what impact gender composition plays in the relationships examined here. Finally, a third limitation of the quantitative study regards the use of a single item measure to assess perceptions of team adaptive performance, which prevented us from determining the construct’s content validity and the reliability (Matthews et al., 2022).

Considering our limitations, we envision three pathways to extend our current work. First, future studies could complement our event-based research through the adoption of non-intrusive methods such as action-cameras or sociometric badges, that could provide more information on how changes in group social structures amidst Antarctica summer and wintering missions correlate with the different team adaptation processes within a specific episode. Likewise, we suggest that future research takes a longitudinal approach to examine how teams modify their team processes over time in the face of different triggers. Both options could, additionally, allow for a deepening of our knowledge of other adaptation trigger attributes including elements of event time such as duration, timing, and strength change (Kennedy & Maynard, 2017).

Second, future work could focus on nationality/language specific facilities and increase the amount of data collected in these settings. Eventually, this strategy might also pave the way for cross-cultural research in extreme environments, to clarify how national culture shapes the relationship between team adaptation processes and adaptive performance. Finally, a third extension of our research could disentangle what adaptation mechanisms might be in place when teams are challenged with unexpected events in extreme environments, and what individual characteristics can influence this relationship. Specifically, researchers could examine how engaging in team process adaptation (Maynard et al., 2015) versus psychological adaptation can be (a) triggered by specific adaptation trigger attributes, (b) contribute to distinct team outcomes, (c) depending on team adaptive expertise (Paletz et al., 2013). For example, internal triggers may activate adaptation processes, while external triggers might lead participants to engage in psychological adaptation (Palinkas & Suedfeld, 2021). Whereas modifying adaptation processes might have direct implications for team performance, engaging in psychological adaptation might contribute to well-being and cohesion (Golden et al., 2018). The decision to engage in either of them can in turn be shaped by team adaptive expertise and the extent to which teams see themselves as capable of engaging with the situation competently, or not.

## Conclusion

Team adaptation forms the cornerstone of effective teamwork, and this impact becomes even more salient for teams performing in dynamic, extreme environments where teams must adapt to changing circumstances often multiple times a day. To thrive in the solitude of a space habitat, the harshness of an offshore platform, or the remoteness of an Antarctic station, teams must be capable of adapting how they work, and, perhaps most importantly, how they plan their work and manage their interpersonal relationships. Furthermore, the modification of these team processes becomes more/less salient in the face of triggers that are more/less critical and emerge from within the team or from the broader environment.

## Data Availability

The datasets generated during and/or analyzed during the current study are available from the corresponding author on reasonable request.

## Appendix

### Interview Guidelines PROPOLAR Mission 2019/20

#### Oral Informed Consent/and Introduction

· Disclose confidentially, anonymity and privacy.
· Explain who accesses to the data and how are we going to use it.
· We would like to record this, is that ok for you?
· Imagine we are aliens and we know nothing about Antarctica and what you are doing here
· There is not right or wrong

#### Demographics

I have some background questions for you. If I may ask:

· How old are you?
· What is your background?
· What is your role here in Antarctica (being Station Chief, Senior Scientist, Logistic, Polar Representative)?
· For how long have you been doing this job?
   ◦ Can you recall on how many field missions you have been enrolled before?
· Before being Station Chief, Senior Scientist, Polar Representative, did you have any other roles or assignments. How did they relate with your current role?

### Effective Teamwork

· How does your team look like?
· In your view, which behaviors make some Antarctica Teams more effective than others?
   ◦ In your mind, what makes teamwork effective in these teams? Any particular elements? And ineffective teamwork?
   ◦ How about your own team, what things you like the most versus the least about working with your fellow team members? Provide examples?
· We have learned from scientists that they very often need the support of station personnel to do their work.
   ◦ When science teams come to you because they need your help, what makes it easier for you to help them? What makes it harder? Provide examples.
· We also learned from scientists that sometimes they come to you with unexpected and challenging situations.
   ◦ Can you please describe a specific situation when a research team came to you with a challenging situation and you and your team had to work with the scientists to ensure that they achieve their mission goals?
   ◦ Please think back to a specific situation when your team experienced a challenging situation and how you work together to resolve it. What were the team members doing that helped to you achieve your goal?
   ◦ Is there anything that if done differently would have made things better?
· We also learned from station managers that when there is an unexpected and challenging situation, you may ask for support from other teams and stations.
   ◦ Please describe your role in that and how do you coordinate with other teams and other stations to help scientists doing their work? Can you recall a particular event that went well and why? Another situation that did not go as well and why?

### Relationship Building

· One of the things we’ve learned during our previous campaign is that how science teams and station personnel get along with each other is important.
   ◦ What contributes to **good/poor** working relationship with the science teams? What helps them develop? Can you provide examples?
   ◦ What contributes to poor working relationships? Can you give an example?

We know that having good working relationships within your team is important. Here in Antarctica, what helps your team developed good working relationships, what gets in the way of developing good working relationships? Does this also have implications for the way you deal with unexpected and challenging situations?

### Planning & Debriefing

· During the summer season many research teams come to this station. From your perspective what kind of planning between your team and the research teams ensures mission success?
· We know that when people discuss how things went, something we call a debriefing, it helps them improve in the future. When and how do you debrief with your team? What is that debriefing look like?

#### Closing Question

· Our interview is coming to an end. Is there anything else you would like to add?
